# Differences in end-of-life care patterns between types of hospice used for cancer patients: a retrospective cohort study

**DOI:** 10.1186/s12904-024-01442-2

**Published:** 2024-04-30

**Authors:** Il Yun, Eun-Cheol Park, Chung Mo Nam, Jaeyong Shin, Suk-Yong Jang, Sung-In Jang

**Affiliations:** 1 Department of Preventive Medicine, Gachon University College of Medicine, Incheon, Republic of Korea; 2 Institute of Health Services Research, Yonsei University, Seoul, Republic of Korea; 3 Department of Preventive Medicine & Institute of Health Services Research, Yonsei University, 50-1 Yonsei-to, Seodaemun-Gu, Seoul, 03722 Republic of Korea; 4 Department of Healthcare Management, Graduate School of Public Health, Yonsei University, Seoul, Republic of Korea

**Keywords:** Hospice, Cancer patients, End-of-life care, Care pattern, Quality of life

## Abstract

**Background:**

In response to the rapid aging population and increasing number of cancer patients, discussions on dignified end-of-life (EoL) decisions are active around the world. Therefore, this study aimed to identify the differences in EoL care patterns between types of hospice used for cancer patients.

**Methods:**

In this population-based cohort study, the Korean National Health Insurance Service cohort data containing all registered cancer patients who died between 2017 and 2021 were used. A total of 408,964 individuals were eligible for analysis. The variable of interest, the type of hospice used in the 6 months before death, was classified as follows: (1) Non-hospice users; (2) Hospital-based hospice single users; (3) Home-based hospice single users; (4) Combined hospice users. The outcomes were set as patterns of care, including intense care and supportive care. To identify differences in care patterns between hospice types, a generalized linear model with zero-inflated negative binomial distribution was applied.

**Results:**

Hospice enrollment was associated with less intense care and more supportive care near death. Notably, those who used combined hospice care had the lowest probability and frequency of receiving intense care (aOR: 0.18, 95% CI: 0.17–0.19, aRR: 0.47, 95% CI: 0.44–0.49), while home-based hospice single users had the highest probability and frequency of receiving supportive care (Prescription for narcotic analgesics, aOR: 2.95, 95% CI: 2.69–3.23, aRR: 1.45, 95% CI: 1.41–1.49; Mental health care, aOR: 3.40, 95% CI: 3.13–3.69, aRR: 1.35, 95% CI: 1.31–1.39).

**Conclusion:**

Our findings suggest that although intense care for life-sustaining decreases with hospice enrollment, QoL at the EoL actually improves with appropriate supportive care. This study is meaningful in that it not only offers valuable insight into hospice care for terminally ill patients, but also provides policy implications for the introduction of patient-centered community-based hospice services.

**Supplementary Information:**

The online version contains supplementary material available at 10.1186/s12904-024-01442-2.

## Introduction

In recent decades, cancer has emerged as the primary cause of mortality worldwide. In 2020, approximately 10 million people had died from cancer, accounting for roughly one-sixth of all recorded deaths [1]. Although notable improvements in cancer detection and treatment have extended the lifespans of many cancer patients, many patients continue to be diagnosed at advanced terminal stages. Moreover, patients requiring advanced care often contend with physical and psychological symptoms stemming from their illnesses, treatment, or concurrent health issues [2]. Regrettably, these symptoms are frequently left unaddressed by conventional medical care, thereby affecting the patients’ well-being and relationships with their families [3–5]. Accordingly, hospice and palliative care initiatives were introduced to enhance the quality of life (QoL) of terminally ill patients and their caregivers by prioritizing relief rather than cure [6].

Hospice and palliative care are integral components of patient-centered healthcare and a part of a global ethical obligation to mitigate profound impacts of severe health conditions, encompassing physical, emotional, and spiritual dimensions [7]. The World Health Organization estimates that approximately 56.8 million individuals, including 25.7 million in their final year of life, require palliative care annually [8, 9]. This demand is rising owing to global aging trends and the increasing prevalence of chronic illnesses such as cancer, heart disease, and dementia. However, the current provision of palliative care falls far short of meeting this need, reaching only approximately 14% of people requiring the services [8, 9].

Furthermore, in response to Korea’s rapidly aging population, deliberations regarding dignified end-of-life (EoL) decisions are ongoing. In 2016, the Korean National Assembly enacted the “Act on Hospice and Palliative Care and Decision on Life-Sustaining Treatment for Patients at End of Life” [10, 11], which permits terminally ill patients to make the choice to forego life-sustaining treatment (LST). In Korea, cancer accounts for one in every four deaths, and approximately 23.2% of all patients with cancer-related deaths are involved in hospice care services [12], indicating LST withdrawal. Although three types of hospice services—hospital-based, home-based, and consultative hospices—have been introduced in Korea, most patients opt for hospital-based hospice care. Only 4% of patients choose home-based hospice care [12], whereas consultative hospice services serve as a supplementary step before patient enrollment in hospital- or home-based hospice care. This current state of hospice utilization in Korea prompted us to consider the efficacy of this policy.

Therefore, in this study, we aimed to identify differences in EoL care patterns among the types of hospices used for cancer patients. We believe that our findings can provide information on the use of hospice care as a means for terminally ill patients to exercise their right to self-determination at the end of their lives.

## Methods

### Data and study population

In this population-based cohort study, we obtained data from the Korean National Health Insurance Service (NHIS) database. Since the implementation of universal health coverage in 1989, all South Korean citizens have been obliged to subscribe to the NHIS, and approximately 98% of the entire population has been enrolled. The NHIS database includes the International Classification of Diseases 10th revision (ICD-10) diagnostic codes, prescriptions for medications, length of hospital stay, medical expenses, and information regarding healthcare provisions [13].

**Fig. 1:**
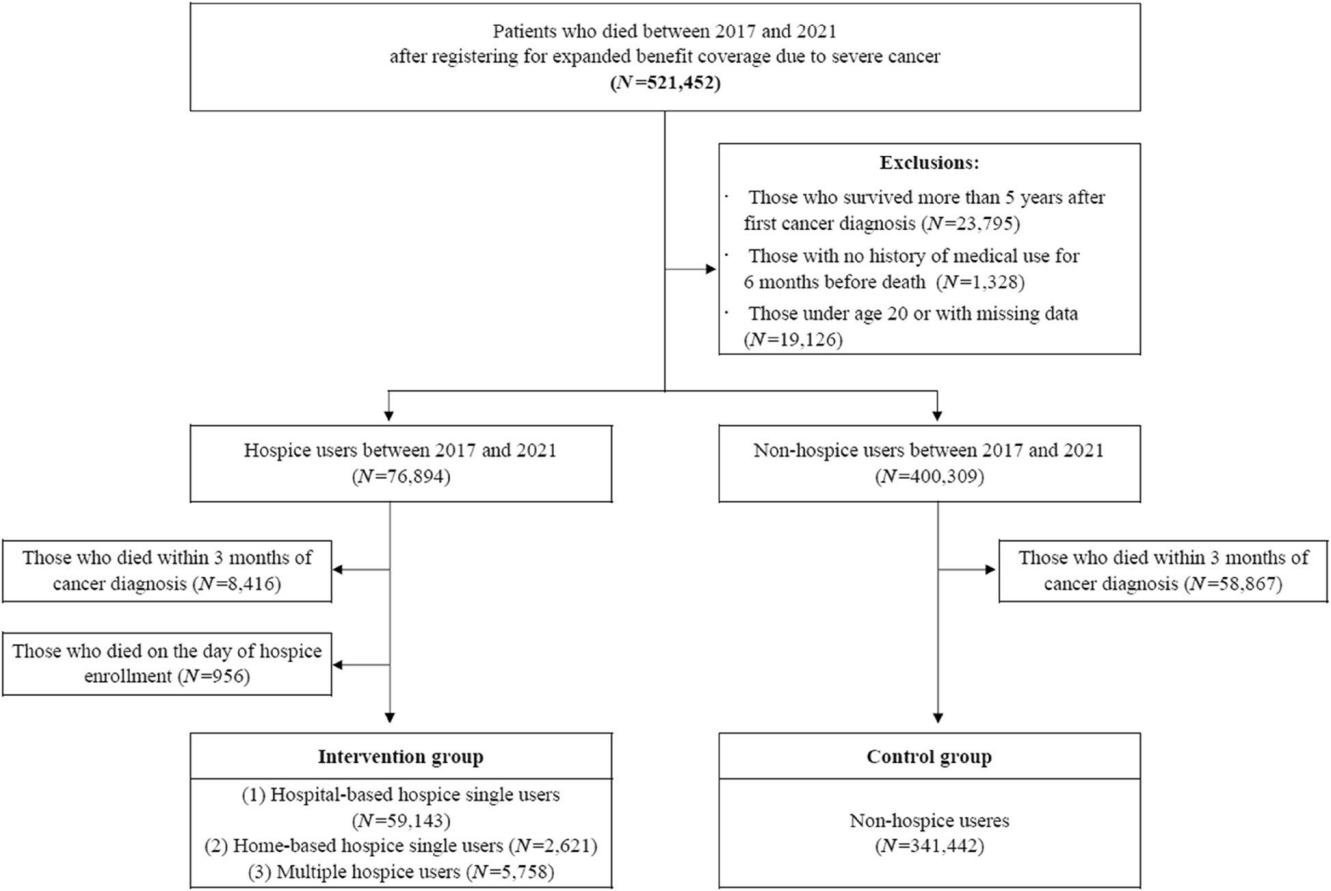
Flow chart of study population selection

To explore the impact of the type of hospice used on cancer patients’ EoL care patterns, we included cancer patients who died after registering for expanded benefit coverage due to severe cancer (claim code: “V193”). Subsequently, we obtained the NHIS cohort data of all 521,452 registered cancer patients who died between January 1, 2017 and December 31, 2021. We excluded those who survived > 5 years after their first cancer diagnosis, had no medical records for 6 months before death, and aged < 20 years or had missing data were sequentially excluded, resulting in a total of 477,203 participants. Among them, 76,894 individuals had used hospice care within 6 months before death, whereas 400,309 did not. Because we aimed to investigate care patterns and expenditures 1 and 3 months before death based on hospice care use, we excluded patients who died within 3 months of their cancer diagnosis. As determining patients who died on the day of hospice enrollment in the intervention group, we also excluded such patients (*n* = 956). Finally, 408,964 individuals were eligible for analysis, of which 67,522 and 341,422 individuals were in the intervention and control groups, respectively. A flowchart of the study sample selection process is shown in Fig. 1.

### Variables

The dependent variables were the patterns of care, which were divided into intense and supportive care. Intense care refers to aggressive treatment to prolong the life of cancer patients [14]; in this study, it was specifically defined as intubation and ventilator use, cardiopulmonary resuscitation, hemodialysis, care in the intensive care unit (ICU), or computed tomography (CT) use. Supportive care refers to pain control and psychological relief management that can significantly impact the cancer patients’ QoL [15–18] and was defined as a prescription of narcotic analgesics and visits to psychiatry and family medicine clinics (Supplementary Table 1). For these outcomes, whether the patient received care in the last 30 and 90 days of life was identified as a binary variable, and the total amount of care was identified as a count variable.

The type of hospice used in the 6 months before death was classified into four categories as follows: those who have (1) never used hospice (reference group); (2) only used hospital-based hospice (claim codes: “WJ-,” “WK-,” “WL-,” “WM-,” “WN-,” “WO-,” “WG-,” “WH-”); (3) only used home-based hospice (claim codes: “AP-”); and (4) used both hospital- and home-based hospice within 6 months before death.

We included 10 variables in the analysis as covariates. First, as sociodemographic factors, sex (males and females), age (range: < 30, 30–39, 40–49, 50–59, 60–69, and ≥ 70 years), region (Seoul and metropolitan cities, small cities, and rural area), income level (low [quintile, 1–6], middle [7–13], high [14–20]) and type of health insurance subscription (regionally-insured, workplace-insured, and Medicaid) were included in the analysis. Second, as factors related health status, we adjusted for the Charlson comorbidity index (CCI) score (range: 0–1 and ≥ 2), primary cancer type (lung, liver, colorectal, gastric, pancreatic, gallbladder/bile duct, breast, and prostate cancer, non-Hodgkin's lymphoma, leukemia, and other types), survival time after cancer diagnosis (90–365 days, 366–730 days, 731–1,095 days, and ≥ 1,096 days), and the year of death.

### Statistical analysis

We used the chi-squared test to examine the distribution of general characteristics of the study population in the year of death. General characteristics are presented as frequencies (n) and percentages (%), whereas descriptive statistics for all dependent variables are reported as means and standard deviations. To identify differences in care patterns between hospice types, a generalized linear model with a zero-inflated negative binomial (ZINB) distribution was applied. Count data containing a large number of zeros are commonly observed across various fields, such as medicine and public health [19, 20]. Zero inflation, which often signifies overdispersion, indicates that the frequency of zero counts exceeds expectations. When the overdispersion in raw data is due to zero inflation, the zero-inflated Poisson (ZIP) model serves as a standard framework for fitting the data [21]. After factoring in zero inflation and if the data persistently indicate further overdispersion, the ZINB model should be considered [22]. This model combines a distribution degenerate at zero with a baseline negative binomial distribution as an alternative to the ZIP model [23, 24]. As a result, the ZINB model had two components [25, 26]: First, we estimated the odds ratios (OR) from a logistic regression model (zero component). Second, we estimated the risk ratio (RR) using the results of the negative binomial regression model (count component). In this study, the zero component was modeled to estimate the probability that an excess zero will not occur, that is, a non-zero probability.

All statistical analyses were conducted using the SAS version 9.4 software (SAS Institute Inc., Cary, NC, USA). Statistical significance was set at *p* < 0.05.

## Results

Table 1 presents the general characteristics of the study population in their year of death. Among all participants, the proportion of patients who were male, aged > 60 years, had high-income, and regionally insured was reported to be high regardless of the type of hospice used. Meanwhile, in the non-hospice user group, more patients were living in small cities or rural areas; however, among home-based hospice only and combined hospice users, many were living in Seoul and metropolitan cities. In addition, among all hospice types, patients with a CCI score of 0–1 and with a survival period > 3 years (1,096 days) were the most frequently reported. Lung and colorectal cancer accounted for the highest proportion of the top 10 primary cancer types. The number of cancer deaths tended to increase every year; however, during the coronavirus disease pandemic in 2020 and 2021, the proportion of patients using only hospital-based and combined hospice services decreased, whereas that of patients who used only home-based hospice and non-hospice users noticeably increased.

**Table 1:** General characteristics of study population in year of death

**Characteristics**	**Type of hospice used**								
	**Hospital-based hospice only**		**Home-based hospice only**		**Combined hospice**		**None**		***P*-value**
	**N**	**%**	**N**	**%**	**N**	**%**	**N**	**%**	
**Total (*N* = 408,964)**	59,143	14.5	2,621	0.6	5,758	1.4	341,442	83.5	
**Sex**									< .0001
Men	34,424	58.2	1,484	56.6	3,002	52.1	216,926	63.5	
Women	24,719	41.8	1,137	43.4	2,756	47.9	124,516	36.5	
**Age (years)**									< .0001
< 30	656	1.1	23	0.9	78	1.4	3,352	1.0	
30 ~ 39	2,348	4.0	84	3.2	229	4.0	8,993	2.6	
40 ~ 49	7,214	12.2	268	10.2	603	10.5	27,969	8.2	
50 ~ 59	14,233	24.1	499	19.0	1,326	23.0	61,860	18.1	
60 ~ 69	16,934	28.6	802	30.6	1,647	28.6	90,559	26.5	
≥ 70	17,758	30.0	945	36.1	1,875	32.6	148,709	43.6	
**Region**									< .0001
Seoul and metropolitan cities	28,505	48.2	1,563	59.6	3,326	57.8	150,814	44.2	
Small cities and rural	30,638	51.8	1,058	40.4	2,432	42.2	190,628	55.8	
**Income level**									< .0001
Low	15,423	26.1	605	23.1	1,273	22.1	95,464	28.0	
Middle	16,823	28.4	691	26.4	1,522	26.4	92,166	27.0	
High	26,897	45.5	1,325	50.6	2,963	51.5	153,812	45.0	
**Health insurance type**									< .0001
Regionally-insured	35,725	60.4	1,698	64.8	3,758	65.3	198,537	58.1	
Workplace-insured	20,812	35.2	836	31.9	1,830	31.8	119,161	34.9	
Medicaid	2,606	4.4	87	3.3	170	3.0	23,744	7.0	
**CCI score**									< .0001
0 ~ 1	53,657	90.7	2,372	90.5	5,200	90.3	317,086	92.9	
≥ 2	5,486	9.3	249	9.5	558	9.7	24,356	7.1	
**Primary cancer type**									< .0001
Lung cancer	5,942	10.0	256	9.8	542	9.4	33,069	9.7	
Liver cancer	4,156	7.0	156	6.0	330	5.7	23,356	6.8	
Colorectal cancer	5,746	9.7	276	10.5	631	11.0	30,266	8.9	
Gastric cancer	5,146	8.7	227	8.7	496	8.6	30,187	8.8	
Pancreatic cancer	3,124	5.3	123	4.7	380	6.6	8,865	2.6	
Gallbladder/bile duct cancer	2,214	3.7	105	4.0	218	3.8	9,558	2.8	
Breast cancer	2,254	3.8	113	4.3	226	3.9	10,018	2.9	
Prostate cancer	1,321	2.2	71	2.7	151	2.6	13,300	3.9	
Non-Hodgkin's Lymphoma	541	0.9	19	0.7	46	0.8	4,391	1.3	
Leukemia	241	0.4	3	0.1	18	0.3	4,186	1.2	
Else	28,458	48.1	1,272	48.5	2,720	47.2	174,246	51.0	
**Survival time after cancer diagnosis (days)**									< .0001
90 ~ 365	16,707	28.2	680	25.9	1,355	23.5	86,287	25.3	
366 ~ 730	13,504	22.8	574	21.9	1,298	22.5	63,715	18.7	
731 ~ 1095	8,331	14.1	377	14.4	871	15.1	43,494	12.7	
≥ 1096	20,601	34.8	990	37.8	2,234	38.8	147,946	43.3	
**Year of death**									< .0001
2017	11,023	18.6	311	11.9	917	15.9	64,170	18.8	
2018	11,808	20.0	388	14.8	1,007	17.5	65,792	19.3	
2019	12,969	21.9	519	19.8	1,294	22.5	66,886	19.6	
2020	11,939	20.2	645	24.6	1,265	22.0	70,579	20.7	
2021	11,404	19.3	758	28.9	1,275	22.1	74,015	21.7	

Descriptive statistics for intense care are presented in Supplementary Table 2, and results of the ZINB regression model exploring the differences in intense care according to the type of hospice used are presented in Table 2. The odds of receiving intense care in the last 30 days of life was significantly lower among hospice users than among non-hospice users. For combined hospice users, the odds was estimated to be as low as 82% (hospital-based hospice only, adjusted OR [aOR]: 0.36, 95% CI: 0.35–0.37; home-based hospice only, aOR: 0.37, 95% CI: 0.34–0.40; combined hospice, aOR: 0.18, 95% CI: 0.17–0.19). Furthermore, analysis using the count model of patients receiving intense care at least once in the last 30 days of life revealed that the number of patients receiving intense care among hospice users was significantly lower than that among non-hospice users. Similar to the results of the logistic model, the difference in the number of patients receiving intense care with that in non-hospice users was the largest in combined hospice users (hospital-based hospice only, adjusted RR [aRR]: 0.57, 95% CI: 0.56–0.58; home-based hospice only, aRR: 0.61, 95% CI: 0.58–0.65; combined hospice, aRR: 0.47, 95% CI: 0.44–0.49). When the outcome was set as intense care during the last 90 days of life, similar results was observed (Supplementary Table 3). The probability of receiving intense care in the last 90 days of life was significantly lower among hospice users, irrespective of hospice type, than among non-hospice users. Significant differences were observed in the total number of ICU visits in the last 90 days, depending on the type of hospice used.

**Table 2:** Differences in intense care in the last 30 days of life according to the type of hospice used

**Variables**	**Intense care in the last 30 days of life**					
	**Zero-inflation (logistic model, non-zero probability)**			**Negative Binomial (count model)**		
	**aOR**	**95% CI**	***P*-value**	**aRR**	**95% CI**	***P*-value**
**Type of hospice used**						
None	ref			ref		
Hospital-based hospice only	0.36	(0.35–0.37)	< .0001	0.57	(0.56–0.58)	< .0001
Home-based hospice only	0.37	(0.34–0.40)	< .0001	0.61	(0.58–0.65)	< .0001
Combined hospice	0.18	(0.17–0.19)	< .0001	0.47	(0.44–0.49)	< .0001
**Sex**						
Men	ref			ref		
Women	0.77	(0.76–0.78)	< .0001	0.95	(0.95–0.96)	< .0001
**Age (years)**						
< 30	ref			ref		
30 ~ 39	0.93	(0.83–1.05)	0.2354	1.28	(1.21–1.35)	< .0001
40 ~ 49	0.82	(0.73–0.91)	0.0002	1.31	(1.25–1.38)	< .0001
50 ~ 59	0.76	(0.69–0.84)	< .0001	1.30	(1.24–1.37)	< .0001
60 ~ 69	0.67	(0.61–0.74)	< .0001	1.35	(1.29–1.41)	< .0001
≥ 70	0.39	(0.35–0.43)	< .0001	1.26	(1.20–1.32)	< .0001
**Region**						
Seoul and metropolitan cities	ref			ref		
Small cities and rural	0.88	(0.87–0.90)	< .0001	0.96	(0.95–0.97)	< .0001
**Income level**						
Low	0.87	(0.86–0.89)	< .0001	0.98	(0.97–0.99)	< .0001
Middle	0.94	(0.92–0.95)	< .0001	0.97	(0.96–0.97)	< .0001
High	ref			ref		
**Health insurance type**						
Regionally-insured	ref			ref		
Workplace-insured	0.98	(0.96–0.99)	0.0006	1.01	(1.00–1.02)	0.0765
Medicaid	0.79	(0.77–0.81)	< .0001	1.09	(1.07–1.11)	< .0001
**CCI score**						
0 ~ 1	ref			ref		
≥ 2	1.01	(0.99–1.04)	0.2728	1.29	(1.27–1.31)	< .0001
**Primary cancer type**						
Lung cancer	ref			ref		
Liver cancer	0.91	(0.88–0.94)	< .0001	1.02	(1.00–1.04)	0.0200
Colorectal cancer	0.73	(0.71–0.76)	< .0001	1.12	(1.10–1.14)	< .0001
Gastric cancer	0.79	(0.77–0.82)	< .0001	1.12	(1.10–1.14)	< .0001
Pancreatic cancer	0.85	(0.82–0.89)	< .0001	0.95	(0.93–0.98)	< .0001
Gallbladder/bile duct cancer	0.89	(0.85–0.93)	< .0001	0.98	(0.95–1.00)	0.0591
Breast cancer	1.00	(0.96–1.05)	0.9659	1.11	(1.09–1.14)	< .0001
Prostate cancer	0.82	(0.79–0.85)	< .0001	1.18	(1.15–1.21)	< .0001
Non-Hodgkin's Lymphoma	1.17	(1.10–1.25)	< .0001	1.33	(1.29–1.38)	< .0001
Leukemia	1.51	(1.41–1.63)	< .0001	1.39	(1.35–1.44)	< .0001
Else	0.90	(0.88–0.92)	< .0001	1.21	(1.19–1.22)	< .0001
**Survival time after cancer diagnosis (days)**						
90 ~ 365	ref			ref		
366 ~ 730	0.98	(0.96–1.00)	0.0196	1.00	(0.99–1.01)	0.8998
731 ~ 1095	0.96	(0.94–0.99)	0.0010	1.06	(1.05–1.07)	< .0001
≥ 1096	1.05	(1.03–1.07)	< .0001	1.18	(1.16–1.19)	< .0001
**Year of death**						
2017	ref			ref		
2018	1.07	(1.05–1.10)	< .0001	1.16	(1.14–1.17)	< .0001
2019	1.14	(1.12–1.16)	< .0001	1.30	(1.28–1.32)	< .0001
2020	1.11	(1.09–1.14)	< .0001	1.44	(1.42–1.46)	< .0001
2021	1.12	(1.10–1.15)	< .0001	1.54	(1.53–1.56)	< .0001

Descriptive statistics for supportive care are presented in Supplementary Table 4, and results of the ZINB regression model exploring the differences in supportive care according to the type of hospice used are presented in Table 3. The odds of receiving a prescription for narcotic analgesics in the last 30 days of life was significantly higher among hospice users than among nonhospice users. Notably, home-based hospice single users had a 2.95 times higher probability of receiving the prescription than non-hospice users (hospital-based hospice only, aOR: 1.19, 95% CI: 1.15–1.22; home-based hospice only, aOR: 2.95, 95% CI: 2.69–3.23; combined hospice, aOR: 1.98, 95% CI: 1.85–2.13). In addition, analysis using a count model of individuals prescribed narcotic analgesics at least one in their last 30 days of life, the total number of prescriptions in hospice users was significantly higher than that in the non-hospice users (hospital-based hospice only, aRR: 1.39, 95% CI: 1.38–1.41; home-based hospice only, aRR: 1.45, 95% CI: 1.41–1.49; combined hospice, aRR: 1.45, 95% CI: 1.42–1.49). Regarding prescriptions for narcotic analgesics in the last 90 days of life, a similar pattern was observed (Supplementary Table 5). A significant difference was observed among the types of hospice used in both the probability of being prescribed narcotic analgesics and total number of prescriptions, with user of only home-based hospice having the highest probability.

**Table 3:** Differences in prescriptions for narcotic analgesics in the last 30 days of life according to the type of hospice used

**Variables**	**Prescriptions for narcotic analgesics in the last 30 days of life**					
	**Zero-inflation (logistic model, non-zero probability)**			**Negative binomial (count model)**		
	**aOR**	**95% CI**	***P*-value**	**aRR**	**95% CI**	***P*-value**
**Type of hospice used**						
None	ref			ref		
Hospital-based hospice only	1.19	(1.15–1.22)	< .0001	1.39	(1.38–1.41)	< .0001
Home-based hospice only	2.95	(2.69–3.23)	< .0001	1.45	(1.41–1.49)	< .0001
Combined hospice	1.98	(1.85–2.13)	< .0001	1.45	(1.42–1.49)	< .0001
**Sex**						
Men	ref			ref		
Women	0.90	(0.88–0.92)	< .0001	0.99	(0.99–1.00)	0.2561
**Age (years)**						
< 30	ref			ref		
30 ~ 39	1.10	(0.96–1.26)	0.1758	0.98	(0.93–1.03)	0.4464
40 ~ 49	0.98	(0.87–1.11)	0.7839	0.96	(0.92–1.01)	0.1095
50 ~ 59	0.89	(0.79–1.01)	0.0695	0.95	(0.91–1.00)	0.0528
60 ~ 69	0.71	(0.63–0.80)	< .0001	0.94	(0.90–0.99)	0.0114
≥ 70	0.40	(0.36–0.46)	< .0001	0.93	(0.88–0.97)	0.0010
**Region**						
Seoul and metropolitan cities	ref			ref		
Small cities and rural	0.87	(0.86–0.89)	< .0001	0.98	(0.97–0.99)	< .0001
**Income level**						
Low	0.89	(0.86–0.92)	< .0001	0.99	(0.98–1.00)	0.0456
Middle	0.92	(0.89–0.94)	< .0001	0.99	(0.98–1.01)	0.3122
High	ref			ref		
**Health insurance type**						
Regionally-insured	ref			ref		
Workplace-insured	0.96	(0.93–0.98)	0.0001	1.00	(0.99–1.01)	0.6488
Medicaid	0.63	(0.60–0.66)	< .0001	0.99	(0.96–1.01)	0.2346
**CCI score**						
0 ~ 1	ref			ref		
≥ 2	1.03	(0.99–1.07)	0.1114	0.99	(0.98–1.01)	0.2447
**Primary cancer type**						
Lung cancer	ref			ref		
Liver cancer	0.70	(0.66–0.73)	< .0001	1.00	(0.98–1.02)	0.7239
Colorectal cancer	0.69	(0.66–0.73)	< .0001	1.00	(0.98–1.02)	0.7068
Gastric cancer	0.59	(0.56–0.62)	< .0001	1.01	(0.99–1.03)	0.1772
Pancreatic cancer	1.07	(1.01–1.13)	0.0266	1.01	(0.99–1.03)	0.4320
Gallbladder/bile duct cancer	0.80	(0.75–0.86)	< .0001	1.00	(0.97–1.02)	0.8113
Breast cancer	0.98	(0.92–1.04)	0.4943	1.02	(1.00–1.05)	0.0686
Prostate cancer	0.61	(0.57–0.66)	< .0001	0.99	(0.96–1.02)	0.4595
Non-Hodgkin's Lymphoma	0.46	(0.41–0.51)	< .0001	0.99	(0.95–1.04)	0.7681
Leukemia	0.32	(0.28–0.37)	< .0001	1.12	(1.06–1.18)	< .0001
Else	0.66	(0.64–0.68)	< .0001	1.01	(1.00–1.02)	0.1538
**Survival time after cancer diagnosis (days)**						
90 ~ 365	ref			ref		
366 ~ 730	1.00	(0.97–1.03)	0.8585	0.99	(0.98–1.00)	0.0397
731 ~ 1095	0.89	(0.86–0.92)	< .0001	0.99	(0.97–1.00)	0.0634
≥ 1096	0.74	(0.72–0.76)	< .0001	0.99	(0.98–1.00)	0.0155
**Year of death**						
2017	ref			ref		
2018	1.03	(1.00–1.07)	0.0839	0.99	(0.97–1.00)	0.1218
2019	1.08	(1.04–1.12)	< .0001	0.99	(0.98–1.01)	0.3243
2020	1.24	(1.20–1.28)	< .0001	1.00	(0.98–1.01)	0.7652
2021	1.32	(1.28–1.37)	< .0001	1.00	(0.98–1.01)	0.7607

Subsequently, we analyzed differences in mental health care before death according to the type of hospice used (Table 4). Compared with non-hospice users, all three types of hospice users had approximately four times higher odds of receiving mental health care in their last 30 days of life (hospital-based hospice only, aOR: 3.58, 95% CI: 3.51–3.66; home-based hospice only, aOR: 4.96, 95% CI: 4.58–5.36; combined hospice, aOR: 4.46, 95% CI: 4.22–4.72). The number of mental health care was also significantly associated with the type of hospice used (hospital-based hospice only, aRR: 1.24, 95% CI: 1.22–1.26; home-based hospice only, aRR: 5.91, 95% CI: 5.56–6.29; combined hospice, aRR: 4.91, 95% CI: 4.69–5.15). Particularly, home-based hospice only users were estimated to have received 5.91 times more mental health care in the last 30 days of life than non-hospice users. Similar results from the logistic model were observed when differences in mental health care in the last 90 days of life were set as outcomes (Supplementary Table 6). Meanwhile, based on the count model, combined hospice users were estimated to have the largest difference in the total number of mental healthcare services in their last 90 days of life compared with non-hospice users.

**Table 4:** Differences in mental health care in the last 30 days of life according to the type of hospice used

**Variables**	**Mental health care in the last 30 days of life**					
	**Zero-inflation (logistic model, non-zero probability)**		**Negative binomial (count model)**			
	**aOR**	**95% CI**	***P*-value**	**aRR**	**95% CI**	***P*-value**
**Type of hospice used**						
None	ref			ref		
Hospital-based hospice only	3.58	(3.51–3.66)	< .0001	1.24	(1.22–1.26)	< .0001
Home-based hospice only	4.96	(4.58–5.36)	< .0001	5.91	(5.56–6.29)	< .0001
Combined hospice	4.46	(4.22–4.72)	< .0001	4.91	(4.69–5.15)	< .0001
**Sex**						
Men	ref			ref		
Women	1.12	(1.10–1.14)	< .0001	0.98	(0.96–1.00)	0.0633
**Age (years)**						
< 30	ref			ref		
30 ~ 39	1.11	(0.95–1.30)	0.1716	0.86	(0.73–1.02)	0.0774
40 ~ 49	1.18	(1.02–1.36)	0.0228	0.86	(0.74–1.00)	0.0568
50 ~ 59	1.25	(1.09–1.44)	0.0019	0.88	(0.75–1.02)	0.0815
60 ~ 69	1.34	(1.17–1.54)	< .0001	0.90	(0.78–1.04)	0.1655
≥ 70	1.84	(1.60–2.11)	< .0001	0.96	(0.83–1.11)	0.5610
**Region**						
Seoul and metropolitan cities	ref			ref		
Small cities and rural	0.95	(0.94–0.97)	< .0001	0.93	(0.92–0.95)	< .0001
**Income level**						
Low	1.07	(1.05–1.10)	< .0001	1.00	(0.97–1.02)	0.8577
Middle	1.01	(0.99–1.03)	0.5203	0.99	(0.97–1.02)	0.5738
High	ref			ref		
**Health insurance type**						
Regionally-insured	ref			ref		
Workplace-insured	1.00	(0.98–1.02)	0.9422	0.99	(0.97–1.01)	0.2052
Medicaid	1.20	(1.16–1.24)	< .0001	1.01	(0.97–1.04)	0.6900
**CCI score**						
0 ~ 1	ref			ref		
≥ 2	0.95	(0.93–0.98)	0.0026	1.03	(0.99–1.06)	0.1297
**Primary cancer type**						
Lung cancer	ref			ref		
Liver cancer	0.82	(0.79–0.86)	< .0001	0.95	(0.91–1.00)	0.0335
Colorectal cancer	1.06	(1.02–1.10)	0.0014	1.06	(1.02–1.10)	0.0063
Gastric cancer	1.02	(0.98–1.05)	0.4058	1.05	(1.01–1.09)	0.0211
Pancreatic cancer	0.96	(0.91–1.01)	0.1427	1.02	(0.97–1.08)	0.4563
Gallbladder/bile duct cancer	0.91	(0.86–0.96)	0.0003	1.04	(0.98–1.10)	0.1553
Breast cancer	0.99	(0.94–1.04)	0.6758	1.04	(0.98–1.10)	0.2155
Prostate cancer	1.15	(1.09–1.20)	< .0001	1.06	(1.01–1.12)	0.0325
Non-Hodgkin's Lymphoma	0.75	(0.69–0.81)	< .0001	0.98	(0.89–1.08)	0.6418
Leukemia	0.51	(0.46–0.57)	< .0001	0.83	(0.72–0.95)	0.0058
Else	0.98	(0.95–1.01)	0.1383	1.03	(1.00–1.06)	0.0822
**Survival time after cancer diagnosis (days)**						
90 ~ 365	ref			ref		
366 ~ 730	1.02	(0.99–1.04)	0.1485	1.00	(0.97–1.02)	0.8720
731 ~ 1095	1.05	(1.02–1.08)	0.0004	1.00	(0.97–1.03)	0.8814
≥ 1096	1.06	(1.04–1.09)	< .0001	1.02	(1.00–1.04)	0.1214
**Year of death**						
2017	ref			ref		
2018	1.04	(1.02–1.07)	0.0011	1.03	(1.00–1.06)	0.0323
2019	1.06	(1.03–1.09)	< .0001	1.03	(1.00–1.06)	0.0243
2020	1.05	(1.03–1.08)	0.0001	1.10	(1.07–1.13)	< .0001
2021	1.07	(1.04–1.10)	< .0001	1.15	(1.12–1.18)	< .0001

## Discussion

In this retrospective cohort study, we examined the differences in care patterns during the last 30 and 90 days of life according to the type of hospice care used. The key findings of this cohort study are summarized as follows: First, hospice use was associated with less intense and more supportive care near death. Notably, combined hospice care users had the lowest probability and frequency of receiving intense care, whereas patients using only home-based hospice had the highest probability and frequency of receiving supportive care. This finding is consistent with those of previous studies reporting that hospice and palliative care are effective in reducing the procedural burden and aggressive care at the EoL [27, 28]. Second, we measured narcotic analgesic prescriptions for pain control and psychiatric consultations for psychological relief as supportive care [29, 30]. As a result, we found that patients who used only home-based hospice care received superior pain and mental health management, predicting that this would improve their QoL during their final days. This has implications similar to those of US studies, which demonstrated that nursing home residents enrolled in hospice care had better pain management than those not enrolled in hospice [31, 32].

Several studies have examined the impact of hospice care on healthcare utilization and costs for terminally ill cancer patients. They suggested that offering hospice care at an earlier stage may reduce unnecessary hospital admissions and healthcare resource utilization [33]. Furthermore, the adoption of hospice care tends to lower medical expenses by preventing unnecessary medical interventions [34]. Hospice care also can effectively manage severe pain and enhances the patients’ overall QoL [35, 36]. In countries with diverse hospice service offers, researchers have explored the outcomes of hospital- and home-based hospice models. Patients who opt for home-based hospice care receive palliative support at their own residences and eventually pass away in a familiar and comfortable environment. Therefore, insurance mandates for home-based hospice care in Korea were recently introduced. However, this mandate leads to limited number of studies assessing the effectiveness of each type of care; and no studies have evaluated whether this policy has been implemented as intended. Moreover, although a significant number of patients use more than one type of hospice care depending on their health status or preferences, the effects of this multiple use on healthcare utilization and health outcomes have never been evaluated.

Although the domestic hospice use rate has increased compared with that in the past, the rate remains only 23.7% as of 2022 [37], which is low compared with that of major countries. Even if patients express their intention to withdraw LST and wish to die in their own home, most receive hospice care in a hospital setting at the EoL because of family recommendations or anxiety about their health conditions. This phenomenon is thought to have occurred because awareness of appropriate EoL care, including hospice care, has not yet been properly established in South Korea. Accordingly, the results of this study can provide valuable information and insights for individuals who are hesitant to use hospice care, thereby enabling them to recognize their autonomy rights. Our findings have important implications for the active promotion and development of established policies. Currently, Korea's hospice-eligible diseases include five diseases, including cancer, which is limited compared with those of other major countries. Considering the continuously increasing mortality rates attributable to chronic and geriatric diseases such as dementia, hospice-eligible diseases should be expanded to ensure a dignified EoL for all patients. In addition, based on the policy trends in major countries, Korea should enhance its efforts in advocating for patient-centered community-based hospice care policies. This can be achieved by identifying places where patients express their preference for EoL care or where they would like to spend their final moments.

This study had certain limitations. First, we obtained NHIS cohort data that only included patients who died between 2017 and 2021 after registering for expanded benefit coverage owing to severe cancer; therefore, we were unable to identify medical utilization records, sociodemographic information, and mortality for patients with diseases other than cancer. Hence, individuals who died from hospice-ineligible diseases were not included in the comparison group. Instead, we included cancer patients who died without using hospice care within the same period as the intervention group as a comparison group. Second, although cancer staging is a very important confounder in evaluating outcomes at the EoL of cancer patients, this information was not included in the data we analyzed. Thus, we had to first select people who died after being diagnosed with cancer and then follow them retrospectively. Third, we attempted to measure the duration of hospice use, yet encountered constraints with the data. While the date of hospice enrollment was recorded, the discharge date was absent. Consequently, we couldn’t ascertain whether patients transitioned to a general ward following hospice enrollment or determine the precise duration of hospice use. Fourth, the NHIS cohort dataset was constructed for administrative purposes; therefore, the ICD-10 codes recorded for health insurance claims may not provide detailed clinical information about the patients’ conditions. Furthermore, potential incomplete coding, which could lead to misclassification or underestimation of the outcomes, remains a concern [38–40]. Finally, we attempted to account for potential factors that could affect EoL care patterns and expenditures in cancer patients, such as primary cancer type, survival time after initial cancer diagnosis, and comorbidities. However, it is important to note that we could not completely eliminate the possible impact of unmeasured variables, which could affect these confounding factors.

## Conclusions

Our findings demonstrated that hospice use is associated with receiving less intense and supportive care at the EoL. Notably, because home-based hospice only users receive better pain management and mental health care, their QoL during the final days is expected to improve. Thus, although intense life-sustaining care decreases with hospice enrollment, QoL at the EoL improves with appropriate supportive care. Based on the policy trends of countries with advanced hospice care, developing patient-centered, community-based hospice care policies is advisable. This policy would offer advantages to both the government, by enabling efficient management of medical resources, and patients, who can assert their autonomy and die with dignity and without suffering.

### Supplementary Information

**Supplementary Material 1.**

## Data Availability

No datasets were generated or analysed during the current study.
