# Supplementary material for: Differences in end-of-life care patterns between types of hospice used for cancer patients: a retrospective cohort study
Source: BMC Palliat Care. 2024 Apr 30;23:111. doi: 10.1186/s12904-024-01442-2 (PMC11061907; doi:10.1186/s12904-024-01442-2)
Supplement: Supplementary file 1 — Supplementary Material 1. [file 12904_2024_1442_MOESM1_ESM.docx]

Supplementary table 1. Identification of primary dependent variables in claims data

| **Variables** | | **Claim codes** |
| --- | --- | --- |
| **Intense care^a^** | |  |
|  | Intubation and ventilator use | "M5859", "M5850", "M5857", "M5858", "M5860" |
|  | CPR | "M5873", "M5874", "M5875 , "M5876", "M5877" |
|  | Hemodialysis | "O7020", "O7021", "O7031", "O7032", "O7033", "O7034" |
|  | ICU care | "AJ001", "AJ003", "AJ010", "AJ020", "AJ100", "AJ200", "AJ300" |
|  | CT use | "HA4-" |
| **Supportive care** | |  |
|  | Prescriptions for narcotic analygesics^b^ | "811", "821", "114" |
|  | Mental health care^c^ | "03", "23" |
| ^a^ Procedure codes extracted from the medical history database | | |
| ^b^ Drug classification codes extracted from the prescription database, including prescription information for Codeine, Fentanyl, Hydrocodone, Oxycodone, Hydromorphone, Tramadol, Morphine, and Pethidine | | |
| ^c^ Medical department codes extracted from the medical history database, code “03” for psychiatric treatment and “23” for family medicine treatment | | |

Supplementary table 2. Descriptive statistics on intense care

| **Variables** | | | **Intense care (times, Mean±SD)** | | | | | | | | | | |
| --- | --- | --- | --- | --- | --- | --- | --- | --- | --- | --- | --- | --- | --- |
|  |  |  | **Overall** | | **Intubation and ventilator use** | | **CPR** | | **hemodialysis** | | **ICU care** | | **CT use** |
| **A. In the last 30 days of life**  **Type of hospice used** | | | |  | |  | |  | |  | |  | |
|  | None | | 22.29 ± 41.36 | | 5.42 ± 16.22 | | 1.33 ± 3.89 | | 4.02 ± 26.67 | | 0.74 ± 5.69 | | 10.76 ± 15.96 |
|  | Hospital-based hospice only | | 7.34 ± 16.19 | | 0.15 ± 2.66 | | 0.02 ± 0.50 | | 0.53 ± 9.52 | | 0.06 ± 1.07 | | 6.57 ± 12.07 |
|  | Home-based hospice only | | 8.06 ± 18.40 | | 0.96 ± 5.45 | | 0.50 ± 2.38 | | 0.57 ± 10.89 | | 0.06 ± 0.93 | | 5.97 ± 12.48 |
|  | Combined hospice | | 3.70 ± 10.16 | | 0.08 ± 1.29 | | 0.04 ± 0.70 | | 0.18 ± 5.37 | | 0.02 ± 0.63 | | 3.38 ± 8.42 |
| **B. In the last 30 days of life**  **Type of hospice used** | | |  | |  | |  | |  | |  | |  |
|  | | None | 45.54 ± 95.26 | | 6.80 ± 21.16 | | 1.33 ± 3.91 | | 11.65 ± 8.25 | | 1.18 ± 9.61 | | 24.59 ± 26.10 |
|  | | Hospital-based hospice only | 26.97 ± 41.43 | | 0.44 ± 4.52 | | 0.03 ± 0.55 | | 2.03 ± 33.19 | | 0.21 ± 2.17 | | 24.27 ± 23.20 |
|  | | Home-based hospice only | 24.73 ± 42.87 | | 1.13 ± 6.35 | | 0.50 ± 2.38 | | 1.79 ± 34.42 | | 0.09 ± 1.12 | | 21.22 ± 23.98 |
|  | | Combined hospice | 17.15 ± 26.89 | | 0.16 ± 2.13 | | 0.04 ± 0.68 | | 0.66 ± 18.57 | | 0.06 ± 1.03 | | 16.22 ± 19.26 |

**Supplementary table 3.** Differences in intense care in the last 90 days of life according to the type of hospice used

| **Variables** | | **Intense care in the last 90 days of life** | | | | | | | | | |
| --- | --- | --- | --- | --- | --- | --- | --- | --- | --- | --- | --- |
|  |  | **Zero-inflation (logistic model,**  **non-zero probability)** | | | | | **Negative Binomial (count model)** | | | | |
|  |  | **aOR** | **95% CI** | | | **P-value** | **aRR** | **95% CI** | | | **P-value** |
| **Type of hospice used** | |  |  |  |  |  |  |  |  |  |  |
|  | None | ref. |  |  |  |  | ref. |  |  |  |  |
|  | Hospital-based hospice only | 0.89 | (0.87 | - | 0.91) | <.0001 | 0.60 | (0.59 | - | 0.60) | <.0001 |
|  | Home-based hospice only | 0.68 | (0.62 | - | 0.74) | <.0001 | 0.58 | (0.56 | - | 0.60) | <.0001 |
|  | Combined hospice | 0.41 | (0.39 | - | 0.43) | <.0001 | 0.48 | (0.46 | - | 0.49) | <.0001 |
| **Sex** |  |  |  |  |  |  |  |  |  |  |  |
|  | Men | ref. |  |  |  |  | ref. |  |  |  |  |
|  | Women | 0.71 | (0.69 | - | 0.72) | <.0001 | 0.91 | (0.91 | - | 0.92) | <.0001 |
| **Age (years)** | |  |  |  |  |  |  |  |  |  |  |
|  | < 30 | ref. |  |  |  |  | ref. |  |  |  |  |
|  | 30 ~ 39 | 1.09 | (0.94 | - | 1.28) | 0.2582 | 1.29 | (1.23 | - | 1.35) | <.0001 |
|  | 40 ~ 49 | 0.87 | (0.76 | - | 1.00) | 0.0536 | 1.28 | (1.23 | - | 1.33) | <.0001 |
|  | 50 ~ 59 | 0.78 | (0.68 | - | 0.89) | 0.0002 | 1.28 | (1.23 | - | 1.33) | <.0001 |
|  | 60 ~ 69 | 0.59 | (0.51 | - | 0.67) | <.0001 | 1.29 | (1.24 | - | 1.34) | <.0001 |
|  | ≥ 70 | 0.24 | (0.21 | - | 0.28) | <.0001 | 1.09 | (1.05 | - | 1.13) | <.0001 |
| **Region** | |  |  |  |  |  |  |  |  |  |  |
|  | Seoul and metropolitan cities | ref. |  |  |  |  | ref. |  |  |  |  |
|  | Small cities and rural | 0.89 | (0.88 | - | 0.91) | <.0001 | 0.94 | (0.94 | - | 0.95) | <.0001 |
| **Income level** | |  |  |  |  |  |  |  |  |  |  |
|  | Low | 0.87 | (0.85 | - | 0.88) | <.0001 | 0.95 | (0.95 | - | 0.96) | <.0001 |
|  | Middle | 0.93 | (0.91 | - | 0.94) | <.0001 | 0.95 | (0.95 | - | 0.96) | <.0001 |
|  | High | ref. |  |  |  |  | ref. |  |  |  |  |
| **Health insurance type** | |  |  |  |  |  |  |  |  |  |  |
|  | Regionally-insured | ref. |  |  |  |  | ref. |  |  |  |  |
|  | Workplace-insured | 0.97 | (0.95 | - | 0.98) | <.0001 | 1.00 | (0.99 | - | 1.01) | 0.9681 |
|  | Medicaid | 0.76 | (0.74 | - | 0.78) | <.0001 | 1.10 | (1.08 | - | 1.11) | <.0001 |
| **CCI score** | |  |  |  |  |  |  |  |  |  |  |
|  | 0 ~ 1 | ref. |  |  |  |  | ref. |  |  |  |  |
|  | ≥ 2 | 1.04 | (1.01 | - | 1.07) | 0.0070 | 1.52 | (1.50 | - | 1.54) | <.0001 |
| **Primary cancer type** | |  |  |  |  |  |  |  |  |  |  |
|  | Lung cancer | ref. |  |  |  |  | ref. |  |  |  |  |
|  | Liver cancer | 0.95 | (0.91 | - | 0.99) | 0.0117 | 1.04 | (1.02 | - | 1.05) | <.0001 |
|  | Colorectal cancer | 0.66 | (0.64 | - | 0.68) | <.0001 | 1.11 | (1.09 | - | 1.12) | <.0001 |
|  | Gastric cancer | 0.76 | (0.73 | - | 0.78) | <.0001 | 1.09 | (1.07 | - | 1.10) | <.0001 |
|  | Pancreatic cancer | 1.08 | (1.03 | - | 1.14) | 0.0030 | 0.95 | (0.93 | - | 0.97) | <.0001 |
|  | Gallbladder/bile duct cancer | 1.08 | (1.02 | - | 1.13) | 0.0039 | 1.00 | (0.98 | - | 1.02) | 0.6852 |
|  | Breast cancer | 0.95 | (0.90 | - | 1.00) | 0.0594 | 1.12 | (1.10 | - | 1.14) | <.0001 |
|  | Prostate cancer | 0.69 | (0.66 | - | 0.73) | <.0001 | 1.15 | (1.13 | - | 1.18) | <.0001 |
|  | Non-Hodgkin's Lymphoma | 1.16 | (1.07 | - | 1.25) | 0.0002 | 1.40 | (1.36 | - | 1.44) | <.0001 |
|  | Leukemia | 1.15 | (1.05 | - | 1.25) | 0.0019 | 1.32 | (1.28 | - | 1.36) | <.0001 |
|  | Else | 0.85 | (0.83 | - | 0.87) | <.0001 | 1.23 | (1.22 | - | 1.24) | <.0001 |
| **Survival time after cancer diagnosis (days)** | | |  |  |  |  |  |  |  |  |  |
|  | 90 ~ 365 | ref. |  |  |  |  | ref. |  |  |  |  |
|  | 366 ~ 730 | 0.99 | (0.97 | - | 1.01) | 0.3696 | 0.99 | (0.98 | - | 1.00) | 0.0118 |
|  | 731 ~ 1095 | 0.91 | (0.89 | - | 0.93) | <.0001 | 1.03 | (1.01 | - | 1.04) | <.0001 |
|  | ≥ 1096 | 0.87 | (0.85 | - | 0.88) | <.0001 | 1.17 | (1.16 | - | 1.18) | <.0001 |
| **Year of death** | |  |  |  |  |  |  |  |  |  |  |
|  | 2017 | ref. |  |  |  |  | ref. |  |  |  |  |
|  | 2018 | 1.08 | (1.05 | - | 1.11) | <.0001 | 1.17 | (1.15 | - | 1.18) | <.0001 |
|  | 2019 | 1.12 | (1.09 | - | 1.15) | <.0001 | 1.35 | (1.33 | - | 1.36) | <.0001 |
|  | 2020 | 1.04 | (1.02 | - | 1.07) | 0.0005 | 1.49 | (1.47 | - | 1.50) | <.0001 |
|  | 2021 | 0.99 | (0.97 | - | 1.01) | 0.3820 | 1.63 | (1.61 | - | 1.65) | <.0001 |

Supplementary table 4. Descriptive statistics on supportive

| **Variables** | | **Supportive care (times, Mean±SD)** | |
| --- | --- | --- | --- |
|  |  | **Prescription of  narcotic analgesics** | **Mental health care** |
| **A. In the last 30 days of life**  **Type of hospice used** | |  |  |
|  | None | 0.48 ± 1.58 | 0.32 ± 0.91 |
|  | Hospital-based hospice only | 0.89 ± 2.41 | 0.70 ± 1.08 |
|  | Home-based hospice only | 1.85 ± 3.30 | 2.88 ± 4.18 |
|  | Combined hospice | 1.41 ± 3.02 | 2.29 ± 3.45 |
| **B. In the last 90 days of life**  **Type of hospice used** | |  |  |
|  | None | 1.40 ± 3.56 | 0.78 ± 2.12 |
|  | Hospital-based hospice only | 3.29 ± 5.32 | 1.31 ± 2.18 |
|  | Home-based hospice only | 4.61 ± 6.00 | 4.77 ± 7.49 |
|  | Combined hospice | 4.63 ± 6.11 | 4.81 ± 6.78 |

**Supplementary table 5.** Differences in prescriptions for narcotic analgesics in the last 90 days of life according to the type of hospice used

| **Variables** | | **Prescriptions for narcotic analgesics in the last 90 days of life** | | | | | | | | | |
| --- | --- | --- | --- | --- | --- | --- | --- | --- | --- | --- | --- |
|  |  | **Zero-inflation (logistic model,**  **non-zero probability)** | | | | | **Negative binomial (count model)** | | | | |
|  |  | **aOR** | **95% CI** | | | **P-value** | **aRR** | **95% CI** | | | **P-value** |
| **Type of hospice used** | |  |  |  |  |  |  |  |  |  |  |
|  | None | ref. |  |  |  |  | ref. |  |  |  |  |
|  | Hospital-based hospice only | 1.98 | (1.94 | - | 2.02) | <.0001 | 1.27 | (1.26 | - | 1.28) | <.0001 |
|  | Home-based hospice only | 3.40 | (3.13 | - | 3.69) | <.0001 | 1.35 | (1.31 | - | 1.39) | <.0001 |
|  | Combined hospice | 3.32 | (3.13 | - | 3.51) | <.0001 | 1.34 | (1.32 | - | 1.37) | <.0001 |
| **Sex** |  |  |  |  |  |  |  |  |  |  |  |
|  | Men | ref. |  |  |  |  | ref. |  |  |  |  |
|  | Women | 0.94 | (0.93 | - | 0.96) | <.0001 | 0.97 | (0.96 | - | 0.98) | <.0001 |
| **Age (years)** | |  |  |  |  |  |  |  |  |  |  |
|  | < 30 | ref. |  |  |  |  | ref. |  |  |  |  |
|  | 30 ~ 39 | 1.17 | (1.06 | - | 1.29) | 0.0024 | 1.01 | (0.97 | - | 1.05) | 0.6451 |
|  | 40 ~ 49 | 1.05 | (0.95 | - | 1.15) | 0.3492 | 1.00 | (0.96 | - | 1.04) | 0.8818 |
|  | 50 ~ 59 | 0.97 | (0.89 | - | 1.06) | 0.5211 | 0.98 | (0.95 | - | 1.02) | 0.4018 |
|  | 60 ~ 69 | 0.73 | (0.67 | - | 0.80) | <.0001 | 0.97 | (0.93 | - | 1.00) | 0.0870 |
|  | ≥ 70 | 0.38 | (0.35 | - | 0.42) | <.0001 | 0.90 | (0.86 | - | 0.93) | <.0001 |
| **Region** | |  |  |  |  |  |  |  |  |  |  |
|  | Seoul and metropolitan cities | ref. |  |  |  |  | ref. |  |  |  |  |
|  | Small cities and rural | 0.91 | (0.89 | - | 0.92) | <.0001 | 0.97 | (0.96 | - | 0.98) | <.0001 |
| **Income level** | |  |  |  |  |  |  |  |  |  |  |
|  | Low | 0.90 | (0.88 | - | 0.92) | <.0001 | 1.02 | (1.01 | - | 1.02) | 0.0012 |
|  | Middle | 0.94 | (0.92 | - | 0.96) | <.0001 | 1.00 | (1.00 | - | 1.01) | 0.3875 |
|  | High | ref. |  |  |  |  | ref. |  |  |  |  |
| **Health insurance type** | |  |  |  |  |  |  |  |  |  |  |
|  | Regionally-insured | ref. |  |  |  |  | ref. |  |  |  |  |
|  | Workplace-insured | 0.95 | (0.94 | - | 0.97) | <.0001 | 1.00 | (0.99 | - | 1.01) | 0.6072 |
|  | Medicaid | 0.63 | (0.61 | - | 0.66) | <.0001 | 0.97 | (0.95 | - | 0.99) | 0.0003 |
| **CCI score** | |  |  |  |  |  |  |  |  |  |  |
|  | 0 ~ 1 | ref. |  |  |  |  | ref. |  |  |  |  |
|  | ≥ 2 | 1.05 | (1.02 | - | 1.08) | 0.0004 | 1.01 | (1.00 | - | 1.02) | 0.2501 |
| **Primary cancer type** | |  |  |  |  |  |  |  |  |  |  |
|  | Lung cancer | ref. |  |  |  |  | ref. |  |  |  |  |
|  | Liver cancer | 0.67 | (0.65 | - | 0.70) | <.0001 | 0.92 | (0.91 | - | 0.94) | <.0001 |
|  | Colorectal cancer | 0.62 | (0.60 | - | 0.65) | <.0001 | 0.96 | (0.94 | - | 0.97) | <.0001 |
|  | Gastric cancer | 0.52 | (0.50 | - | 0.54) | <.0001 | 0.95 | (0.93 | - | 0.96) | <.0001 |
|  | Pancreatic cancer | 1.23 | (1.17 | - | 1.28) | <.0001 | 1.03 | (1.01 | - | 1.05) | 0.0017 |
|  | Gallbladder/bile duct cancer | 0.83 | (0.79 | - | 0.88) | <.0001 | 0.95 | (0.93 | - | 0.97) | <.0001 |
|  | Breast cancer | 0.81 | (0.77 | - | 0.85) | <.0001 | 1.03 | (1.01 | - | 1.05) | 0.0080 |
|  | Prostate cancer | 0.57 | (0.54 | - | 0.60) | <.0001 | 0.97 | (0.95 | - | 1.00) | 0.0202 |
|  | Non-Hodgkin's Lymphoma | 0.43 | (0.40 | - | 0.47) | <.0001 | 0.90 | (0.87 | - | 0.94) | <.0001 |
|  | Leukemia | 0.26 | (0.24 | - | 0.29) | <.0001 | 0.96 | (0.91 | - | 1.01) | 0.0989 |
|  | Else | 0.63 | (0.61 | - | 0.65) | <.0001 | 0.97 | (0.96 | - | 0.98) | <.0001 |
| **Survival time after cancer diagnosis (days)** | | |  |  |  |  |  |  |  |  |  |
|  | 90 ~ 365 | ref. |  |  |  |  | ref. |  |  |  |  |
|  | 366 ~ 730 | 0.98 | (0.96 | - | 1.01) | 0.1289 | 0.99 | (0.98 | - | 0.99) | 0.0028 |
|  | 731 ~ 1095 | 0.88 | (0.86 | - | 0.90) | <.0001 | 0.97 | (0.96 | - | 0.98) | <.0001 |
|  | ≥ 1096 | 0.72 | (0.70 | - | 0.73) | <.0001 | 0.95 | (0.94 | - | 0.96) | <.0001 |
| **Year of death** | |  |  |  |  |  |  |  |  |  |  |
|  | 2017 | ref. |  |  |  |  | ref. |  |  |  |  |
|  | 2018 | 1.06 | (1.03 | - | 1.08) | <.0001 | 1.00 | (0.99 | - | 1.01) | 0.6573 |
|  | 2019 | 1.11 | (1.08 | - | 1.14) | <.0001 | 1.00 | (0.99 | - | 1.01) | 0.9493 |
|  | 2020 | 1.21 | (1.18 | - | 1.24) | <.0001 | 1.01 | (1.00 | - | 1.02) | 0.2522 |
|  | 2021 | 1.24 | (1.21 | - | 1.27) | <.0001 | 1.01 | (1.00 | - | 1.02) | 0.0194 |

**Supplementary table 6.** Differences in mental health care in the last 90 days of life according to the type of hospice used

| **Variables** | | **Mental health care in the last 90 days of life** | | | | | | | | | |
| --- | --- | --- | --- | --- | --- | --- | --- | --- | --- | --- | --- |
|  |  | **Zero-inflation (logistic model,**  **non-zero probability)** | | | | | **Negative binomial (count model)** | | | | |
|  |  | **aOR** | **95% CI** | | | **P-value** | **aRR** | **95% CI** | | | **P-value** |
| **Type of hospice used** | |  |  |  |  |  |  |  |  |  |  |
|  | None | ref. |  |  |  |  | ref. |  |  |  |  |
|  | Hospital-based hospice only | 3.32 | (3.26 | - | 3.38) | <.0001 | 1.06 | (1.05 | - | 1.08) | <.0001 |
|  | Home-based hospice only | 4.33 | (4.00 | - | 4.68) | <.0001 | 3.48 | (3.28 | - | 3.70) | <.0001 |
|  | Combined hospice | 4.28 | (4.06 | - | 4.52) | <.0001 | 3.64 | (3.49 | - | 3.79) | <.0001 |
| **Sex** |  |  |  |  |  |  |  |  |  |  |  |
|  | Men | ref. |  |  |  |  | ref. |  |  |  |  |
|  | Women | 1.13 | (1.11 | - | 1.14) | <.0001 | 1.05 | (1.03 | - | 1.06) | <.0001 |
| **Age (years)** | |  |  |  |  |  |  |  |  |  |  |
|  | < 30 | ref. |  |  |  |  | ref. |  |  |  |  |
|  | 30 ~ 39 | 1.18 | (1.04 | - | 1.33) | 0.0118 | 1.05 | (0.92 | - | 1.20) | 0.4802 |
|  | 40 ~ 49 | 1.22 | (1.08 | - | 1.37) | 0.0009 | 1.01 | (0.89 | - | 1.15) | 0.8499 |
|  | 50 ~ 59 | 1.26 | (1.12 | - | 1.40) | <.0001 | 1.04 | (0.92 | - | 1.17) | 0.5512 |
|  | 60 ~ 69 | 1.34 | (1.20 | - | 1.50) | <.0001 | 1.04 | (0.92 | - | 1.18) | 0.5157 |
|  | ≥ 70 | 1.82 | (1.63 | - | 2.03) | <.0001 | 1.16 | (1.03 | - | 1.31) | 0.0184 |
| **Region** | |  |  |  |  |  |  |  |  |  |  |
|  | Seoul and metropolitan cities | ref. |  |  |  |  | ref. |  |  |  |  |
|  | Small cities and rural | 0.98 | (0.96 | - | 0.99) | 0.0005 | 0.98 | (0.97 | - | 1.00) | 0.0323 |
| **Income level** | |  |  |  |  |  |  |  |  |  |  |
|  | Low | 1.05 | (1.03 | - | 1.07) | <.0001 | 1.00 | (0.98 | - | 1.02) | 0.7312 |
|  | Middle | 0.98 | (0.97 | - | 1.00) | 0.0432 | 0.98 | (0.96 | - | 0.99) | 0.0084 |
|  | High | ref. |  |  |  |  | ref. |  |  |  |  |
| **Health insurance type** | |  |  |  |  |  |  |  |  |  |  |
|  | Regionally-insured | ref. |  |  |  |  | ref. |  |  |  |  |
|  | Workplace-insured | 1.00 | (0.98 | - | 1.01) | 0.7536 | 0.99 | (0.98 | - | 1.01) | 0.2984 |
|  | Medicaid | 1.16 | (1.13 | - | 1.19) | <.0001 | 1.13 | (1.09 | - | 1.16) | <.0001 |
| **CCI score** | |  |  |  |  |  |  |  |  |  |  |
|  | 0 ~ 1 | ref. |  |  |  |  | ref. |  |  |  |  |
|  | ≥ 2 | 1.19 | (1.14 | - | 1.24) | <.0001 | 1.03 | (1.00 | - | 1.06) | 0.0357 |
|  |  |  |  |  |  |  |  |  |  |  |  |
| **Primary cancer type** | |  |  |  |  |  |  |  |  |  |  |
|  | Lung cancer | ref. |  |  |  |  | ref. |  |  |  |  |
|  | Liver cancer | 0.80 | (0.77 | - | 0.82) | <.0001 | 0.93 | (0.89 | - | 0.96) | <.0001 |
|  | Colorectal cancer | 0.98 | (0.95 | - | 1.01) | 0.2480 | 1.08 | (1.04 | - | 1.11) | <.0001 |
|  | Gastric cancer | 0.98 | (0.95 | - | 1.02) | 0.3231 | 1.05 | (1.02 | - | 1.08) | 0.0034 |
|  | Pancreatic cancer | 0.98 | (0.94 | - | 1.03) | 0.4409 | 1.01 | (0.97 | - | 1.06) | 0.6418 |
|  | Gallbladder/bile duct cancer | 0.89 | (0.85 | - | 0.93) | <.0001 | 1.00 | (0.96 | - | 1.05) | 0.8518 |
|  | Breast cancer | 0.98 | (0.93 | - | 1.03) | 0.3785 | 1.02 | (0.97 | - | 1.07) | 0.4265 |
|  | Prostate cancer | 1.10 | (1.05 | - | 1.14) | <.0001 | 1.10 | (1.05 | - | 1.15) | <.0001 |
|  | Non-Hodgkin's Lymphoma | 0.72 | (0.67 | - | 0.77) | <.0001 | 0.88 | (0.81 | - | 0.95) | 0.0013 |
|  | Leukemia | 0.51 | (0.47 | - | 0.56) | <.0001 | 0.82 | (0.74 | - | 0.91) | 0.0002 |
|  | Else | 0.97 | (0.95 | - | 1.00) | 0.0267 | 1.04 | (1.02 | - | 1.07) | 0.0010 |
| **Survival time after cancer diagnosis (days)** | | |  |  |  |  |  |  |  |  |  |
|  | 90 ~ 365 | ref. |  |  |  |  | ref. |  |  |  |  |
|  | 366 ~ 730 | 1.02 | (1.00 | - | 1.04) | 0.1151 | 1.02 | (0.99 | - | 1.04) | 0.1598 |
|  | 731 ~ 1095 | 1.06 | (1.04 | - | 1.09) | <.0001 | 1.02 | (1.00 | - | 1.05) | 0.0562 |
|  | ≥ 1096 | 1.08 | (1.07 | - | 1.10) | <.0001 | 1.07 | (1.05 | - | 1.09) | <.0001 |
| **Year of death** | |  |  |  |  |  |  |  |  |  |  |
|  | 2017 | ref. |  |  |  |  | ref. |  |  |  |  |
|  | 2018 | 1.06 | (1.04 | - | 1.09) | <.0001 | 1.03 | (1.01 | - | 1.05) | 0.0141 |
|  | 2019 | 1.08 | (1.06 | - | 1.11) | <.0001 | 1.04 | (1.02 | - | 1.06) | 0.0006 |
|  | 2020 | 1.08 | (1.06 | - | 1.10) | <.0001 | 1.06 | (1.03 | - | 1.08) | <.0001 |
|  | 2021 | 1.11 | (1.08 | - | 1.13) | <.0001 | 1.09 | (1.07 | - | 1.11) | <.0001 |
